# Introducing FRED: Software for Generating Feedback Reports for Ecological Momentary Assessment Data

**DOI:** 10.1007/s10488-023-01324-4

**Published:** 2024-01-10

**Authors:** Aljoscha Rimpler, Björn S. Siepe, Carlotta L. Rieble, Ricarda K. K. Proppert, Eiko I. Fried

**Affiliations:** 1 Department of Psychometrics and Statistics, University Groningen, Groningen, Netherlands; 2 Psychological Methods Lab, Department of Psychology, Philipps-University Marburg, Marburg, Germany; 3 Department of Clinical Psychology, Leiden University, Leiden, Netherlands

**Keywords:** Ecological Momentary Assessment, Experience Sampling Method, Shiny App, Personalized Feedback

## Abstract

Ecological Momentary Assessment (EMA) is a data collection approach utilizing smartphone applications or wearable devices to gather insights into daily life. EMA has advantages over traditional surveys, such as increasing ecological validity. However, especially prolonged data collection can burden participants by disrupting their everyday activities. Consequently, EMA studies can have comparably high rates of missing data and face problems of compliance. Giving participants access to their data via accessible feedback reports, as seen in citizen science initiatives, may increase participant motivation. Existing frameworks to generate such reports focus on single individuals in clinical settings and do not scale well to large datasets. Here, we introduce FRED (Feedback Reports on EMA Data) to tackle the challenge of providing personalized reports to many participants. FRED is an interactive online tool in which participants can explore their own personalized data reports. We showcase FRED using data from the WARN-D study, where 867 participants were queried for 85 consecutive days with four daily and one weekly survey, resulting in up to 352 observations per participant. FRED includes descriptive statistics, time-series visualizations, and network analyses on selected EMA variables. Participants can access the reports online as part of a Shiny app, developed via the R programming language. We make the code and infrastructure of FRED available in the hope that it will be useful for both research and clinical settings, given that it can be flexibly adapted to the needs of other projects with the goal of generating personalized data reports.

**Supplementary Information:**

The online version contains supplementary material available at 10.1007/s10488-023-01324-4.

## Generating Feedback Reports for Ecological Momentary Assessment Data

In the last decade, hardware and software developments have provided psychologists with new ways to collect data. A common way to do so is using ecological momentary assessments (EMA), where participants are queried repeatedly on their smartphones and other data sources such as wearable devices are used to collect data in participants’ everyday environment (Shiffman et al., 2008). Typical EMA surveys include self-reported moods, thoughts, behaviors, context variables/situational factors, and symptoms (Ebner-Priemer & Trull, 2009). EMA has been used in various settings, including observational (see, e.g., Shiffman et al., 2008), interventional research (Henry et al., 2022), and to collect data used for personalized feedback in clinical settings (Bringmann et al., 2021; Burger et al., 2022; von Klipstein et al., 2023).

The widespread use of EMA is in part due to its advantages, including reduced recall bias through real-time assessment, the possibility to assess dynamic and complex processes through repeated measures in daily life, and the identification of individual stressors and person-environment interactions (Bringmann et al., 2021; Ebner-Priemer & Trull, 2009; Hamaker & Wichers, 2017; Leertouwer et al., 2021). As a result of the aforementioned factors, EMA increases ecological validity, a measure of how generalizable findings are to real-life settings (Mestdagh & Dejonckheere, 2021).

### Compliance in EMA Studies

One particular challenge EMA studies face is participant compliance, as repeated measurements can be burdensome because they interrupt daily activities (Rintala et al., 2019). Reduced compliance results in more missing data, which may bias reported experiences and behaviors because they often depend on context (Shiffman et al., 2008). For example, participants may be less likely to complete a survey when they are severely distressed compared to a neutral mood, or when they are in a bar with friends compared to when they are home alone. As a result, certain types of thoughts, feelings, behaviors, or situations can be systematically underrepresented in the resulting data. Therefore, increasing the compliance of participants can improve the quality of research results.

Multiple solutions have been proposed to increase compliance; they can broadly be categorized into study design, extrinsic rewards, and intrinsic rewards (Doherty et al., 2020). First, study design choices such as querying people about their experiences at fixed measurement prompts (i.e., exactly at noon every day) can increase compliance. Because participants may adjust their behavior to these schedules (i.e., avoid starting activities shortly before prompts), this may reduce ecological validity (Rintala et al., 2019). Second, extrinsic rewards such as monetary reimbursement have been shown to be effective but can come at considerable costs, especially in large scale projects. Therefore, using intrinsically motivating rewards may be a promising third option to increase compliance. Several studies have shown that intrinsic rewards such as real-time feedback are an effective way to increase compliance, likely independently from design and reimbursement choices (Bälter et al., 2012; Hsieh et al., 2008). Feedback reports may also increase the quality of the data, given that participants motivated by receiving a report may be more interested in filling out questionnaires carefully.

### Feedback Reports on EMA Data

In most cases, researchers in psychology collect and own data. Participants are unable to read resulting publications due to journal paywalls, and they also do not have access to their own data. Citizen science projects focus on increasing stakeholder involvement. One way to do so, which aligns with efforts to increase compliance, is to share data with participants via personalized reports.

Here we introduce and showcase the *Feedback Reports on EMA Data* (FRED) framework, a software tool we developed to generate personalized feedback reports for around 2,000 participants in the WARN-D study who participate in a three-month EMA data collection period with up to 352 measurement points. We understand feedback as a way to grant participants access to their data in a summarized form to provide them with an overview of their psychological functioning during the study period (Leertouwer et al., 2021). Nonetheless, it is important to note that providing insights to participants could serve as an unintended intervention, a point we elaborate more thoroughly in the “Discussion” section of this paper (see also: Fried et al., 2023).

Our primary objective was to develop a tool that allows the communication of EMA data to participants in a way that is understandable and insightful. Contrary to other feedback reports (e.g., von Klipstein et al., 2023), the feedback provided here was not intended as part of an intervention, but rather as a means of providing insights to participants. Feedback reports can take different forms, like downloadable static/animated report files or interactive apps participants can access online (e.g., Bringmann et al., 2021). After creating an initial feedback prototype using html files in the WARN-D study, we progressed to the development of FRED. FRED is an interactive online Shiny app (Chang et al., 2021) that enables participants to explore their data interactively. Currently, available open source feedback tools (e.g., ESMvis by Bringmann et al., 2021) are designed to provide feedback in clinical settings for a small number of participants, and are not easy to implement for large scale studies. Tools for larger samples also exist (e.g., How Nuts are the Dutch by Blaauw et al., 2014), but they are not open source. With FRED we provide a feedback tool that is open source, usable in large scale studies, and useful in both clinical and non-clinical settings.

FRED is developed in the R programming environment (R Core Team, 2022) and can be accessed using a web browser (https://solo-fsw.shinyapps.io/FRED/). Participants need a user key with which they can access parts of their own data via various summary statistics and visualizations of time-series data, including network models that show the dynamic interplay of variables. We provide a guest user key on the log-in page to access the full Shiny app with example data. The R-code and all supplementary materials are available online (https://osf.io/8q254/).

## Methods

### Sample

This work is part of the WARN-D project (Fried et al., 2023), aiming to build a personalized early warning system for depression. The WARN-D research team follows around 2,000 higher-education students (in four cohorts of 500 participants each) over two years to gain a better understanding of stressors and experiences students face that could ultimately lead to mental health problems. The research consists of three stages: a baseline survey, an EMA phase, and a follow-up phase. In the current work, data from the EMA phase of the first two cohorts of WARN-D participants are used. During the EMA phase spanning 85 days, participants were asked to complete four questionnaires per day and an additional questionnaire each Sunday (the codebook is available in the supplementary materials of the WARN-D protocol paper (Fried et al., 2023; see https://osf.io/2jd9h/)). The surveys included both closed and open-ended questions. Furthermore, participants were provided with Garmin vivosmart 4 smartwatches to track activity data; these data will not be analyzed for the current personalized feedback reports.

Inclusion and exclusion criteria for the WARN-D study, as well as detailed information on reimbursement, are provided elsewhere (Fried et al., 2023). For stage 2, participants received up to 45 € for completing the EMA phase, depending on the number of completed surveys. The study protocol of WARN-D was approved by the ethics committee of the European Research Council and the Psychology Research Ethics Committee at Leiden University (No. 2021-09-06-E.I.Fried-V2-3406).

In total, 484 participants were recruited for the first cohort and 518 for the second cohort. Out of the total of 1002 participants that completed the baseline, 880 (cohort 1: 442, cohort 2: 438) started the EMA phase. For more information about the sample see the supplementary material. Participants in the first and second cohorts received survey prompts from December 6, 2021, through February 28, 2022, and from June 6, 2022, through August 29, 2022, respectively.

### Procedure

Participants used the Ethica data app for Android or iOS (Ethica Data Services Inc., 2021) on their smartphones to receive four prompts per day at semi-random times; the exact regimen can be found in the supplementary material. On Sundays, an additional survey included questions about the previous week. This resulted in both daily and weekly survey patterns. Further information about the different surveys is described elsewhere (Fried et al., 2023). Ethica surveys and FRED reports were available in Dutch and English and could be accessed by users in their preferred language.

### Item and Analyses Selection

In the WARN-D study, personalized reports were created with two principles in mind. First, they should cover enough information to be insightful for participants. Second, they should not be overwhelming, interfere with the purpose of the study (an observational study in which no clinical information like a diagnosis is given to participants), or cause harm. Therefore, we had to decide what information to include in the personalized data reports by selecting variables (what items are the reports based on) and statistical models (what analyses are presented).

#### Variable Selection

In an iterative process, we selected the EMA variables to be included based on the two principles described above. We included variables that we deemed interesting to most participants, and excluded items strongly related to psychopathology, such as suicidal ideation and non-suicidal self-injury. Furthermore, we did not include qualitative EMA data in the reports (e.g., best, and worst events of each day), because such open text fields may contain highly sensitive information related to harmful events that we did not feel comfortable to feed back to participants in our reports; such information is better suited to be included in feedback reports that, for instance, clinicians obtain and then can discuss with clients in person. The final variables included in the reports can be found in the supplementary material.

#### Model Selection

We aimed to use simple models and visualizations that we tried to explain in plain language, omitting statistical details to ensure that the reports are as easy as possible to understand. We developed the final wording of the explanations through iterative discussions within the research group. The Shiny app shows twelve tabs that include explanations of the data, statistics, and data visualizations. Participants can select different variables for which they want to receive information. Overall, the report can be categorized into five main parts (see “Results” section for corresponding figures and details).

First, we provide meta-information, such as definitions of the variables participants can select throughout the report, and information on how many prompts were completed by the participant. Second, we display scatter plots for all continuous variables, including the means of these variables across the entire EMA phase. Third, we present categorical data as bar charts, depicting relative frequencies. Both the scatter plots and bar charts offer a comprehensive overview of all variables available for selection throughout the reports and summarize all data points.

Fourth, we show variables over time using scatter plots. Participants can select variables to be plotted from our pre-selected variables. If the data availability is sufficient, these figures include trendlines. For the visualizations of these trendlines, LOESS (locally estimated scatterplot smoothing) functions are particularly suitable because they can estimate trends without specific shape specifications (Jacoby, 2000). This flexibility allows LOESS to be applied to unknown datasets, including idiographic time-series. This is achieved by using numerous subsets of the data, estimating a trend in each, and then combining the results; the size of the subset is determined by a smoothing parameter. The goal is to find a smoothing parameter that does not ‘smooth over’ meaningful trends to make them disappear but at the same time takes out small random variations in the data. For the piloted reports we selected a smoothing parameter that worked best for most participants; in the Shiny app we used the same default but enabled participants to select their own smoothing parameter. When data availability is too low to create smoothed trendlines, the results are just scatterplots.

Fifth, we implement network analyses, which have previously been used to generate feedback in clinical settings (e.g., Hall et al., 2022; von Klipstein et al., 2023). Due to power concerns, we only estimate and show these networks for participants who completed more than 50% of the relevant surveys (following Mansueto et al., 2022). We estimate two lag-1 vector-autoregressive network models (Epskamp et al., 2018), one for all observations 4 times a day (where lag-1 is ~ 4 h), and one for all evening observations (where lag-1 is 24 h). These models are regularized partial correlation models. For the reports, we used contemporaneous networks that depict undirected relations at the same measurement occasion. More information on network analyses, as well as imputation of time-series data using the Kalman Filter (Moritz & Bartz-Beielstein, 2017), is available in the supplementary material. For the analyses, we pre-selected variables that are most normally distributed on an individual level and thus most suitable for the network analyses.

Network analysis estimates many parameters, and there are plausible concerns about the accuracy of network estimation in time series data (Mansueto et al., 2022). For that reason, in our study, FRED only estimates networks when participants have no more than 50% missing data, and we instruct participants to interpret the networks carefully. Nonetheless, to investigate the accuracy of the estimated networks, we conducted network analyses for the 496 participants with sufficient data including the negative affect items overwhelmed, stressed, and sad, and the positive affect items happy, motivated, and relaxed. It is plausible to expect that the majority of relations among negative affect variables should be positive; the majority of relations among positive affect variables should be positive; and the majority of relations among positive and negative affect variables should be negative. We expect to find this in data in case networks are reasonably accurately estimated, and if the precision of our network models would be very low, and edges just based on chance, we would expect different results. We calculated the proportions between positive and negative relations and found that only 0.8%, 9.4%, and 0.5% of the three types of relations (negative-negative, positive-positive, positive–negative) deviated from plausible expectations. Of note, many of the 9.4% negative edges between positive affect states were found between the specific variables relaxed (i.e., calm, low arousal) and motivated (i.e., active, high arousal), which appears plausible to us.

### Feedback on the FRED Prototype

The development of the current version of FRED followed a 3-step process. First, we created a prototype using static html files and pre-selected variables. Second, we asked participants for feedback on this version of the reports. Following this, we developed the Shiny app as a third step. Of 880 participants, 76 participants (8.6%) completed a feedback survey on this FRED prototype; to increase the sample size, we also add data from the 19 participants who completed this survey in cohort 3, leading to a total of n = 95. For more detailed information on this prototype and the feedback survey, see Rimpler (2022). These participants completed an average of 256 EMA surveys (median = 272, min = 57, max = 349). On a scale of 1 to 7 (higher numbers being more favorable feedback), most participants found that the reports described them well (*M* = 5.2, median = 5, *SD* = 1.0) and indicated to understand the reports well (*M* = 5.4, median = 6, *SD* = 1.3). On average, participants reported that the personalized data reports were moderately insightful (*M* = 4.5, median = 5, *SD* = 1.6). Reactions to the reports were on average more positive than negative (*M* = 4.8, median = 5, *SD* = 1.0). Moving to two items where the middle point 4 is most favorable, participants rated the level of detail and length of the reports as close to exactly right (for both, M = 3.8, median = 4, SD = 1.0), rather than, e.g., not enough (1) or too much (7). More detailed information on the items and this survey is available in the supplementary material and Rimpler (2022).

Additionally, we asked participants in open text fields for their reactions to the reports and general feedback. Two of our team members independently looked at these responses and identified common themes. Both raters concluded that in general we received a lot of positive comments, but also some criticism. The positive comments were mainly about the insightfulness and reflection moments of participants when reading the reports. The negative comments were mainly about some additional information that participants would like to receive (e.g., implementation of smartwatch data) or some parts of the report that were not entirely clear to them, sections we consequently updated in later versions of FRED. We also asked participants which sections of the data reports they found most interesting. A figure on this can be found in the supplementary material. Based on this feedback, we decided to continue developing a Shiny app to give participants more control over the information they want to receive. We now describe the development of the application in detail.

### FRED Shiny App

The Shiny app FRED was developed to provide a framework to generate a large amount of personalized data reports. An overview of the R packages we used in the Shiny app can be found in the supplementary material. The code for the Shiny App and a file that explains the functionality and gives a brief tutorial on how to use FRED is provided on OSF (https://osf.io/8q254/).

The most important difference between the FRED prototype described above and the final Shiny app is that all variables were pre-selected in the prototype, whereas participants can dynamically select variables for time-series visualizations and network models in the Shiny app. Additionally, participants can view the individual trajectories of mood states, which were only available as composite scores (e.g., all individual negative affect items were summed into a negative affect score) in the prototype reports.

## Results

### Data Availability

**Fig. 1:**
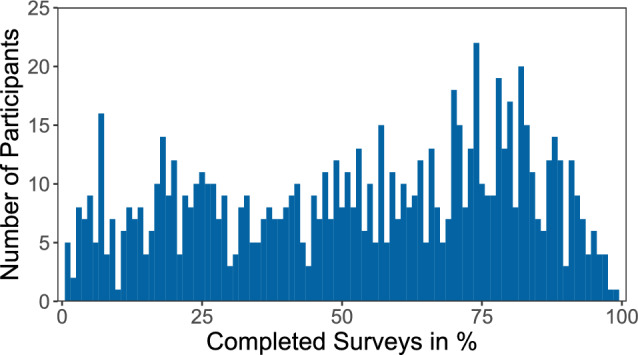
Distribution of data availability. The x-axis shows the number of completed surveys in percent and the y-axis shows the counts in bins of width 5. The distribution has a maximum of 22 participants completing 74% of the surveys

Of the total 352 EMA time points, participants completed an average of 204 (58%) of the surveys. The median of completed surveys was 236 (67%), with a range of 1% to 99% of surveys completed across participants. Figure 1 shows the distribution of data availability per participant.

### Sections Included in the Final Personalized Data Reports

The Shiny app can be accessed via https://solo-fsw.shinyapps.io/FRED/. We provide a guest user key on the log-in page to access the full Shiny app with example data. These example data are based on data from a real participant that has been modified so that the person is not identifiable. All figures in this manuscript are based on the same example data.

The app starts with a login page, explanations of what it entails and how to read it, and a detailed explanation of all items participants can choose to explore. This is followed by the core content, which we describe in the following. For all detailed text blocks, we refer the reader to the example data online (https://solo-fsw.shinyapps.io/FRED/); here, we focus on the most important cornerstones of the report rather than reproducing the reports in full.

#### Completed Surveys

**Fig. 2:**
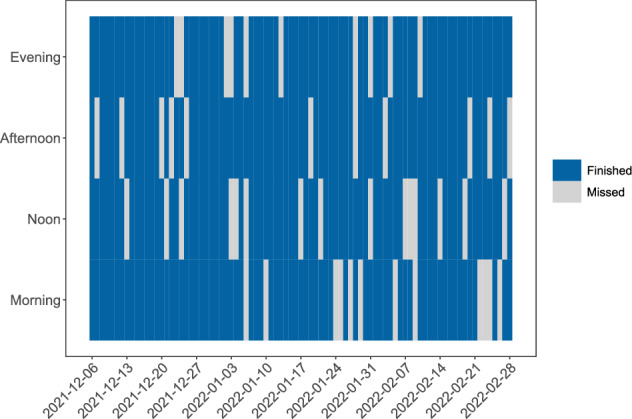
Missing data. This figure indicates which of the daily surveys were completed (blue) or missed (grey). X- and y-axes show the date and time the survey was sent, respectively. This participant completed nearly all surveys and missed more surveys toward the end of the study

In this section, we provide participants with information on how many, and which surveys they completed, both in text form and in the form of a heatmap (Fig. 2).

#### Overview Continuous Data

**Fig. 3:**
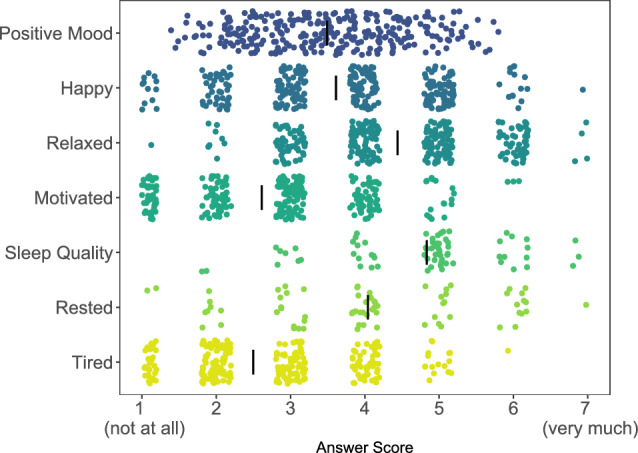
Item distributions and averages. This figure shows a summary of variables relating to positive mood and sleep (y-axis) collected in the morning (sleep quality, rested) or four times per day (remaining variables). The x-axis shows answer scores on a Likert Scale from 1 (not at all) to 7 (very much). Item means are indicated as black vertical bars; this participant reports overall high sleep quality and low feelings of being tired

We present participants with a visual overview of the raw data for continuous variables through scatterplots, which also include the means of these variables. To enhance visibility, individual data points are jittered, allowing for a clearer display of the frequency of specific answer selections. We provide three different plots that summarize data related to positive affect and sleep (Fig. 3), negative affect, and more global items. These scatterplots cover all variables that participants can choose to explore for subsequent tabs, i.e., time-series visualizations and network computations. Participants also receive information on which day, and during which prompts (morning, noon, afternoon, or evening) they had the most positive affect on average.

#### Overview Categorical Data

**Fig. 4:**
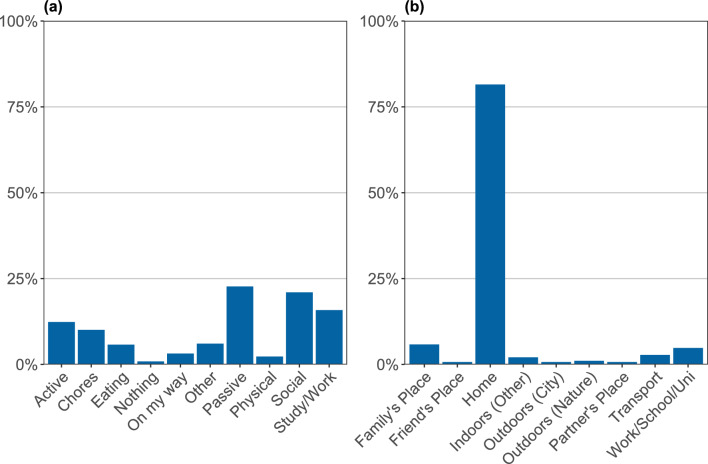
Summary of context items. Participants were queried about their context four times a day. This figure shows a summary for activities (panel **a**) and locations (panel **b**). The x-axis indicates activities/locations, and the y-axis shows relative frequencies of how often participants selected these. This participant most often engaged in social and passive leisure activities (e.g., watching TV); doing nothing was the least frequently endorsed activity. Most often this participant was at home when completing the surveys

**Fig. 5:**
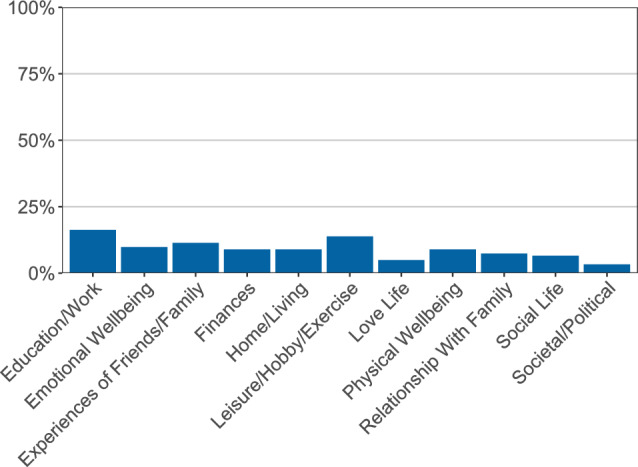
Summary of most negative events. Participants were queried about their most negative event of the day in the evenings. This figure shows a summary of these answers. Items indicating the category to which an event belongs are shown on the x-axis, and the y-axis indicates the relative frequency of how often participants indicated these. The event categories are quite evenly endorsed, with the fewest negative events belonging to the category societal/political and most negative events belonging to the category education/work

We also collected contextual data, including location (where are you right now), if participants were engaging in online or offline social activities, as well as information about their most positive and negative events. These data are summarized as relative frequencies and displayed in bar plots. Examples for activities, locations and negative events are displayed in Figs. 4 and 5.

#### Time-Series

**Fig. 6:**
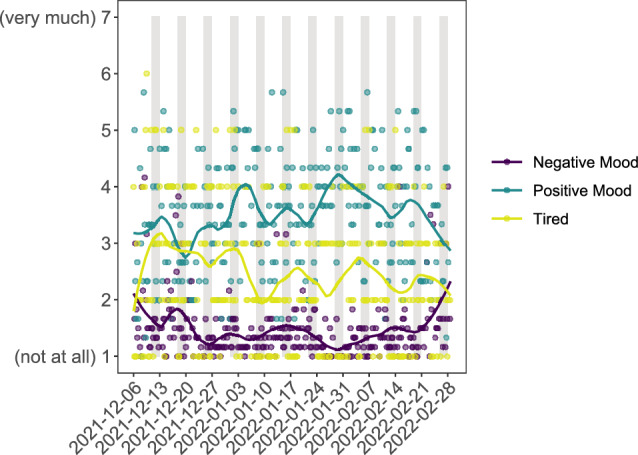
Time-series of tiredness, positive and negative mood. Participants were queried four times a day about their current affective state, which was plotted as time-series. Weekends are highlighted as gray bars. The x-axis shows dates, and the y-axis shows scores on a Likert scale from 1 (not at all) to 7 (very much). For this participant, all three variables show some fluctuation. The person always scored higher on positive than negative mood. Feeling tired often follows a similar trend as positive mood, whereas the trend of negative mood seems to be less connected to positive mood, showing sometimes similar trends (e.g., week 5 and 6) and sometimes opposing trends (e.g., week 3)

We also create three time series graphs so participants can explore the temporal dynamics of their responses. Participants can choose from a set of variables that were recorded four times per day, once every evening, and every Sunday, respectively. Variables cover fluctuations in mood (Fig. 6); daily experiences such as the ability to concentrate, overcome challenges, and their sense of connectedness; and more global items like well-being, stress, and life satisfaction. For a full overview of which variables participants could explore interactively, see the supplementary material. The figures contain raw data points and a smoothed trendline; weekends are highlighted to showcase potential differences between weekdays and weekends.

#### WARN-D Recap

Additionally, we created the *WARN-D* Recap, which provides participants with information about the one week in which they report the most and least positive mean affect, respectively. Participants can also manually zoom in on individual weeks. When a week is selected, we provide participants with information about the most positive and most negative average mood, and average positive/negative mood over the whole data collection period as a comparison. Furthermore, we include week-specific versions of Figs. 4, 5, and 6.

**Fig. 7:**
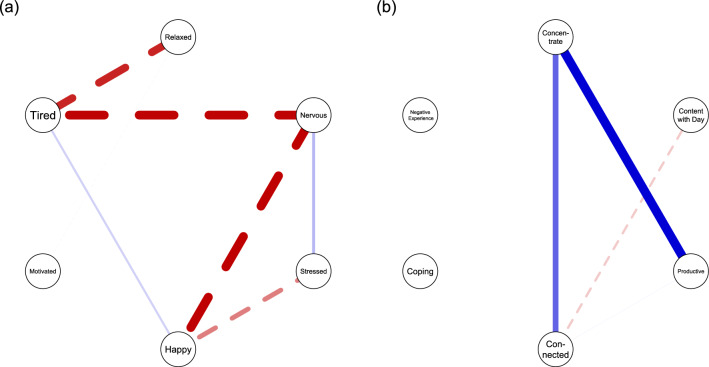
Contemporaneous networks. This figure shows contemporaneous networks of partial correlations between six variables. Positive partial correlations are indicated by blue edges, negative ones by red-dashed edges. The edge thickness indicates the strength of the relations. For the daily network in panel **a**, two positive (e.g., nervous—stressed) and four negative (e.g., tired—nervous) associations were found. For the evening network in panel **b**, two positive edges and one negative edge emerged. Some results are intuitive, while others may initially appear surprising, such as the negative edges between feeling nervous and tired (panel **a**) or feeling less content on days the person feels more connected (panel **b**). There are many potential explanations for this. For example, nervous and tired represent opposite ends on the arousal spectrum, and perhaps feeling connected means that the person engaged in more social activities during the day, but ignored their homework, making them feel less productive in the evening

#### Network Analyses

Finally, we show participants personalized networks. We provide two different personalized networks: the daily and evening networks, which provide information on items queried four times a day or once every evening, respectively (Fig. 7). Both are contemporaneous networks indicating lag-1 controlled partial correlations. Due to power reasons, we only show networks for participants who completed more than 50% of the relevant surveys. Participants can choose the variables for the network analyses, which are the same as for the time-series visualizations. This is the most sophisticated statistical analysis, and we provided detailed explanations of networks, using common terms such as “relationships” instead of “partial correlations”, by omitting specific numerical information, and by using an accessible example (e.g., when you were sad, you were often tired at the same time).

## Discussion

In this paper, we discuss the need for a scalable personalized data report system to increase motivation and compliance in EMA research designs. We aim to tackle the gap of an open source software tool to generate interactive data reports for large scale EMA studies by introducing FRED. We also see our effort of making data accessible and understandable to participants as part of ongoing citizen science initiatives (Tauginienė et al., 2020). Currently, FRED is implemented as a Shiny app to provide participants with an interactive overview of their own data. We queried participants what they thought about FRED. In a feedback survey on our prototype, many participants indicated that they resonated well with the reports, understood the reports, and even gained some new insights. We make all code available so that FRED can be adjusted flexibly by other research teams and hope it will be useful for both research and clinical contexts.

However, creating such reports is not without challenges, and we briefly discuss some of the technical, methodological, and ethical challenges in the upcoming section. First, in observational online research such as the WARN-D study, where participants do not receive the report under supervision, special care must be taken to ensure that participants do not experience potentially harmful consequences from the reports. While assessing clinical constructs such as suicidality does not appear to increase suicidal behavior (DeCou & Schumann, 2018), there is less research on providing feedback to participants (especially in online settings), and there is evidence that receiving false information as feedback can lead to a worsening of symptoms (van Helvoort et al., 2020). To minimize potential harm, we did not include variables that could be used to infer psychopathological states to avoid iatrogenic effects; this was based on the iterative process for variable selection described in the “Methods” section.

Second, feedback can change the thoughts, feelings, and behaviors of participants and, depending on the study design, may serve as an unintended and uncontrolled intervention. For example, if participants receive feedback about the association of negative mood with their social media use, they may change their social media usage. In the WARN-D study, this is a considerable challenge and was difficult to mitigate other than by thinking carefully about which variables to include in the report. We discuss this challenge for the core goal of WARN-D, building an early warning system for depression, in detail elsewhere (Fried et al., 2023).

Third, does one show raw or already analyzed data to participants? While model output such as correlations between variables can provide additional insights, many analyses require prior knowledge to properly interpret them (Bringmann et al., 2021). We tackled this challenge in the WARN-D study by relying largely on basic descriptive statistics and giving simple explanations for more complex statistics.

Fourth, a technical challenge is to determine the medium through which reports are delivered, such as static or animated report files that can be downloaded, or information people can access online, e.g., via interactive apps (e.g., Bringmann et al., 2021). In the WARN-D study, after some initial trial and error using html files, we developed FRED as an interactive online Shiny app (Chang et al., 2021).

Finally, privacy is a concern, specifically when reports are delivered online through an interactive website as implemented for FRED. To address this challenge, participants receive a private, encrypted key to access their reports, which are hosted on university servers. More importantly, all reports are pseudonymized and do not contain identifiable (e.g., demographic, clinical, or qualitative) data so that participants cannot be identified even if reports became public.

### Strengths and Limitations

The current work adds to the field of EMA studies by covering a different use case than other available feedback tools, with a focus on providing personalized reports to hundreds or thousands of users. Since the software is written in R, others can adjust FRED to fit their individual project needs using our annotated code. Furthermore, FRED can be used in a wide variety of different contexts, including both observational and interventional studies, and in research and clinical settings. FRED was developed as part of the WARN-D study with a specific (student) sample in mind, and researchers applying FRED in other contexts and samples should consider adapting the information displayed in the app (e.g., to ensure that the language is appropriate for the sample).

One crucial limitation is that our study design did not actually allow us to answer the question of whether FRED improved motivation and compliance, and reduced attrition, given that we did not have a group of participants that did not have the possibility to obtain reports. Further experimental work about strategies to increase compliance is required before firm conclusions can be drawn. Another limitation is that we only obtained feedback on the pilot, a prototype of FRED. It is possible that introducing some features such as choosing variables may have influenced the comprehensibility of the personalized data reports and thus should be evaluated in future work. For that reason, FRED is under continuous development and evaluation, so that we can respond to potential future concerns by participants to the long list of features we have planned.

### Future Directions

FRED can be extended in various ways; here we briefly discuss extensions regarding data, modeling, and visualizations. Many of the discussed extensions were also explicitly asked for by participants in the feedback survey on the piloted reports, using open-ended text fields.

Regarding data extensions, the WARN-D study also contains qualitative data collected via open text fields. Such qualitative information could be included via *wordclouds* (Fellows, 2018), which indicate the frequency of particular words or phrases used in open text and have been used in supervised EMA feedback before (e.g., Bos et al., 2022). We decided to not provide feedback on open-ended questions because of the challenging nature of some of the participant responses (e.g., containing highly clinical information) that cannot easily be controlled for a system like FRED that aims to scale well to thousands of participants. One approach to tackle this challenge could be to exclude topics from wordclouds that are potentially harmful such as abuse, suicide, and death. Further data extensions of the reports could be smartwatch data we collected, such as activity and sleep. The same guidelines as for self-report data should be followed such as data is understandable for participants and they do not experience harm (e.g., misinterpreting heartbeat data). Analyses could range from mere descriptive statistics to more complex analyses, and different data sources could be combined.

Regarding model extensions, temporal network models could be included in reports (Epskamp et al., 2018). In temporal networks, associations of variables with themselves and with other variables across time are depicted. As opposed to the contemporaneous network, associations between variables are directed, indicating the temporal direction of associations. While this could be a particularly insightful analysis for participants and has been used in clinical feedback settings (Frumkin et al., 2021), we decided not to include these networks for two main reasons. First, directed relations between variables may invoke causal interpretations of the associations in the network for participants in situations where causal inference is not warranted. Second, the results of a temporal network crucially depend on the time frame between surveys, which would add another layer of complexity to the already complicated network visualizations.

Finally, researchers have started moving towards animated time-series graphs (for examples, Bringmann et al., 2021; von Klipstein et al., 2023) which deal well with dynamic features of data (Heer & Robertson, 2007). They can be visually appealing but do not always include additional information. Thus, the use of animations can make information more (Heer & Robertson, 2007) or less accessible (Kriglstein et al., 2012). We opted for static figures because we deemed them more interpretable.

In conclusion, our study highlights the importance of scalable and personalized data report systems in EMA research designs. We showcased FRED as an open-available software tool to create interactive and understandable data reports. We hope FRED contributes to the field by offering a customizable framework to generate personalized data reports for thousands of participants in clinical and research settings.

### Supplementary Information

Below is the link to the electronic supplementary material.

Supplementary file1 (ZIP 305 KB)
