# Supplementary material for: Introducing FRED: Software for Generating Feedback Reports for Ecological Momentary Assessment Data
Source: Adm Policy Ment Health. 2024 Jan 10;51(4):490–500. doi: 10.1007/s10488-023-01324-4 (PMC11196357; doi:10.1007/s10488-023-01324-4)
Supplement: Supplementary file 1 — Supplementary file1 (ZIP 305 KB) [file 10488_2023_1324_MOESM1_ESM.zip › Supplementary Material/Supplement.pdf]

Supplement

Table of Contents

Supplement 1
Sample Description 2
Survey Schedule 2
Items Included in Reports 3
Parameters for Network Analyses 6
Items in Feedback-Survey 6
Interest in Sections of FRED Prototype 7
Used R Packages 8

Sample Description

Variable	Participants not Providing Feedback	Participants Providing Feedback
Gender	F = 652<br>M = 126<br>Other = 24<br>Not shared = 6	F = 89<br>M = 13<br>Other = 3
Age	M = 22.0 (SD = 3.82, range = 18-61)	22.39 (SD = 3.75, range = 18-42)
User Language*	EN = 367<br>NL = 407<br>n.a. = 4	EN = 27<br>NL = 78
Education pursued	MBO = 24<br>HBO Bachelor = 81<br>HBO Master = 11<br>University Bachelor = 373<br>University Master = 271<br>Other = 10	MBO = 7<br>HBO Bachelor= 9<br>University Bachelor = 38<br>University Master = 48<br>Other = 3
Compliance*	48%	65%

Participants providing feedback on their report differed significantly on compliance ($t = -6.53$, $p < .001$), and were more often using the Dutch version ($z = -4.08$, $p < .001$). Participants completing the feedback survey did not differ on the other demographic variables ($p > 0.25$).

Survey Schedule

Survey Name	Time Schedule	Availability	Prompt Pattern	Number of Items
Morning Survey	9:49 - 10:19	20 min	Daily	21 (19 +2 cond.)
Noon Survey	13:34 - 14:04	20 min	Daily	18 (16 +2 cond.)
Afternoon Survey	17:19 - 17:49	20 min	Daily	18 (16 +2 cond.)
Evening Survey	21:04 - 21:34	20 min	Daily	36 (33 + 2 cond. + 1 opt.)
Sunday Survey	11:45 - 12:15	10 h	Weekly	46 (44 + 1 cond. + 1 opt.)

Surveys were sent out at a time in the indicated time frames following a normally distributed jitter; cond.= conditional item (depending on the answer to another item), opt.= optional item; table also in supplementary material of XXX et al. (2023)

Items Included in Reports

Variable Name	Item	Scale
Concentrate	Today, I was able to concentrate and focus well.	*
Connected	Today, I felt connected to other people.	*
Emotion Regulation	Today, it was difficult to cope with my emotions.	*
Enjoyment of Activity	I am enjoying what I am doing right now.	*
Enjoyment Offline Contact	I am enjoying my company.	*
Enjoyment Online Activity	I am enjoying this online activity.	*
Life Satisfaction	All things considered, I am satisfied with my life as a whole.	*
Mental Wellbeing	This week, my overall mental health and emotional wellbeing were:	**
Negative Mood	I feel sad right now.<br>I feel stressed right now.<br>I feel overwhelmed right now.<br>I feel nervous/anxious right now.<br>I am experiencing negative thoughts right now.<br>I feel annoyed/irritated right now.	Composite Score of individual items on * scale
Negative Experience	This event/experience was …<br>Follow up on: ‘My most negative event/experience today was:’	*
Outlook Tomorrow	I am looking forward to tomorrow.	*
Outlook Today	I am looking forward to the rest of the day.	*
Overcome Challenges	I was able to handle today’s challenges well.	*
Physical Discomfort	Today, I experienced physical discomfort/pain.	*
Physical Wellbeing	My overall physical health this week was:	**
Positive Mood	I feel relaxed right now.<br>I feel motivated right now.<br>I feel happy/cheerful right now.	Composite Score of individual items on * scale
Positive Experience	This event/experience was …<br>Follow up on: ‘My most positive event/experience today was:	*
Productive	Today, I felt productive/useful.	*

Variable Name	Item	Scale
Rested	When I woke up, I felt well rested.	*
Satisfaction With Day	Overall, I’m content with how my day went.	*
Sleep Quality	Last night, I slept well.	*
Tired	I feel tired right now.	*
Weekly Stress	This week was stressful for me.	*
Location	Right now, I am at:	home<br>friend's place<br>my family's place<br>work/school<br>transport/public transport<br>other indoors<br>outdoors city<br>outdoors nature
Activity	Right now, my activity is (choose all that apply):	social (offline or online)<br>physical (e.g. cycling, gym)<br>active leisure (hobby, board game)<br>passive leisure (e.g. watching TV, scrolling Instagram)<br>studying/working<br>chores (e.g. cleaning house)<br>on my way to somewhere<br>Eating<br>Other<br>nothing
Online Contact	social online contact<br>right now, I am interacting with others online (choose all that apply):	No<br>Yes: reading/ scrolling/liking<br>Yes: posting<br>Yes: written chatting<br>Yes: call/videocall<br>Yes: doing something with others (e.g.online gaming)

Variable Name	Item	Scale
Offline Contact	Social offline contacts<br>Right now, I am with (choose all that apply):	friend(s)<br>acquaintance(s)/loose contact(s)<br>family<br>romantic partner<br>classmates/co-workers<br>strangers<br>a pet<br>no one
Positive Events	This event/experience belongs to the category (choose all that apply):	social<br>love life<br>personal life<br>education/work<br>home<br>leisure/hobby/pleasure<br>relationship with family<br>Experiences of friends/family<br>Societal/political<br>other
Negative Events	This event/experience belongs to the category (choose all that apply):	social<br>love life<br>personal life<br>education/work<br>home<br>leisure/hobby/pleasure<br>relationship with family<br>Experiences of friends/family<br>Societal/political<br>other

One asterisks (*) indicates that this item was answered on a 7-point Likert scale from '1: Not at all' to '7: Very much'. Two asterisks (**) indicate a scale from '-3: very negative' to '3: very positive".

Parameters for Network Analyses

To avoid false positive network edges, we use the least absolute shrinkage and selection (LASSO) parameter λ. Furthermore, we use the tuning parameter γ (Chen & Chen, 2008) to control the sparseness of the networks. We chose a γ of 0 to increase sensitivity. To ensure data are stationary and meet the assumption for the network models we detrended the data using a linear trend (Mansueto et al., 2022). Because the combination of a Kalman filter for the imputation of missing data and LASSO has been shown to perform well with similar numbers of observations (Mansueto et al., 2022), this combination of methods was chosen for the network analyses.

Items in Feedback-Survey

**Item**	**Scale**
Overall, the report describes me well.	1: Not at all,<br>7: Very Much
I learned something new based on the report.	1: Not at all,<br>7: Very Much
The report is understandable.	1: Not at all,<br>7: Very Much
The amount of information was…	1: Not enough,<br>4: Exactly right,<br>7: Too much
The length of the report was…	1: Not enough,<br>4: Exactly right,<br>7: Too much
The reactions I had when reading the report were…	1: Very negative,<br>7: Very positive
Could you explain your answer, e.g. what reactions you had? (optional)	Open text

Item	Scale
How interesting did you find the different section in your report?	Ranking with most interesting section at top and least interesting at bottom; Only sections the participant received were shown
Do you have any additional feedback for us? Any feedback is welcome, e.g. what you liked, didn't like, or if there were things you would have wanted us to report.	Open text

Interest in Sections of FRED Prototype

In the feedback survey on the pilot version of FRED, we also asked participants to rate the different sections in the personalized data reports according to their interest. Participants were asked to rank each section in a list, with the top of the list representing the most interesting section and the bottom of the list representing the least interesting section.

The figure below shows how participants ranked the sections. Participants were generally most interested in the time series sections and the mood network (renamed to daily network in the shiny app). This was followed by the general summary and the evening network. The summary seems to be almost evenly distributed across the ranks with a tendency to be in the lower ranks. The positive and negative events were most often ranked low. Furthermore, participants were least interested in the

information about the number of completed surveys.

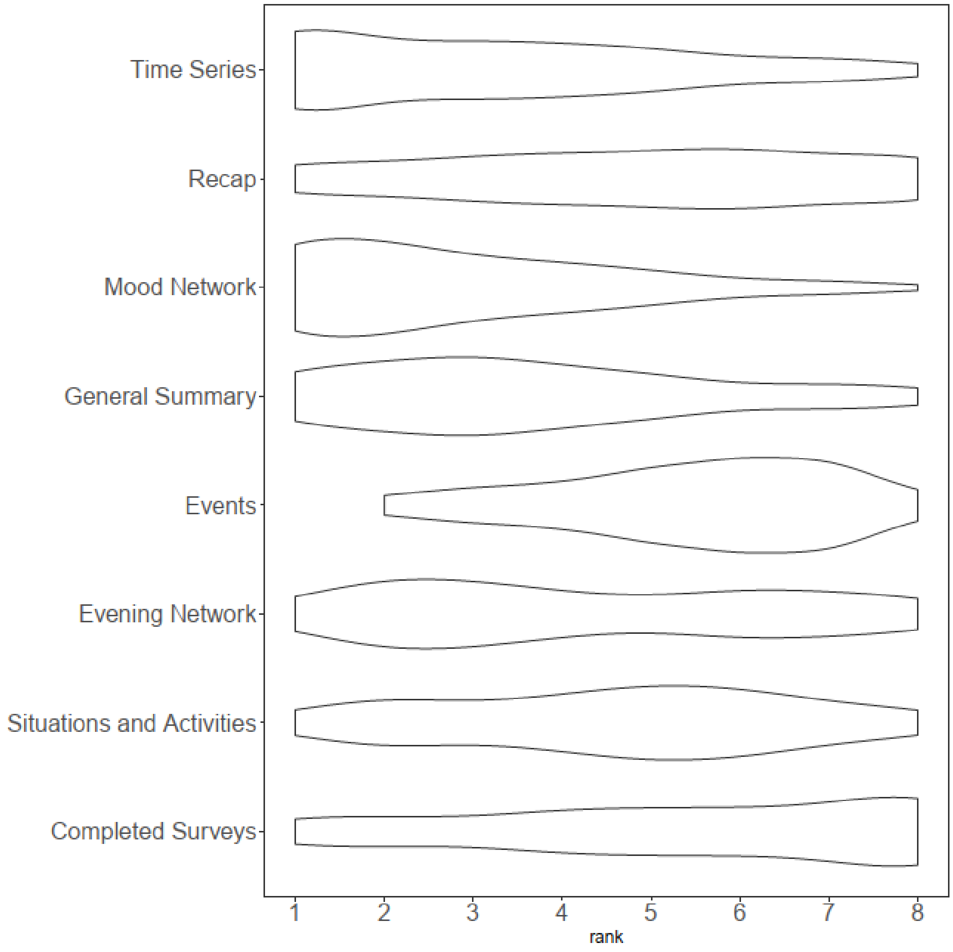

Used R Packages

Multiple R packages are used for FRED to simplify data wrangling, processing of timestamps, analyses, visualizations, and file management. To simplify file management and to be independent of absolute working directories, the *here* package (Müller, 2020) was used. For data wrangling, the package *dplyr* (Wickham et al., 2021) was used. For the processing of timestamps, we used the package *lubridate* (Grolemund et al., 2021). Relative frequencies were calculated with the package *summarytools* (Comtois, 2021). All figures but the networks in the reports are generated using *ggplot2* (Wickham, 2016) and *jtools* (Long, 2020). We used a color

selection for figures that is colorblind-friendly using the package *viridis* (Garnier et al., 2021). For figure alignment. the package *cowplot* (Wilke, 2020) was used. We estimated networks for participants having completed more than 50% of the surveys. To do so we imputed missing data for the selected variables using a Kalman filter (Moritz & Bartz-Beielstein, 2017). Missingness of data was assessed using the *naniar* package (Di Cook et al., 2021) and the Kalman filter imputation was accomplished using *imputeTS* (Moritz & Bartz-Beielstein, 2017). For the network estimation, the package *graphicalVAR* (Epskamp, 2021) was used. The estimated networks were displayed using *qgraph* (Epskamp et al., 2012). For programming the Shiny app the package shiny (Chang et al., 2021) was used. Furthermore, we made use of *shinyjs* (Attali, 2021), *shinyBS* (Bailey, 2022), and *shinyccssloaders* (Sali & Attali, 2020) for the user interface of the Shiny app. To improve the performance of the app, we used the *fst* (Klik, 2022) and *data.table* (Dowle & Srinivasan, 2022) packages.

References

Attali, D. (2021). *shinyjs: Easily Improve the User Experience of Your Shiny Apps in Seconds*. https://CRAN.R-project.org/package=shinyjs

Bailey, E. (2022). *shinyBS: Twitter Bootstrap Components for Shiny*. https://CRAN.R-project.org/package=shinyBS

Chang, W., Cheng, J., Allaire, J. J., Sievert, C., Schloerke, B., Xie, Y., Allen, J., McPherson, J., Dipert, A., & Borges, B. (2021). *shiny: Web Application Framework for R*.

Chen, J., & Chen, Z. (2008). Extended Bayesian information criteria for model selection with large model spaces. *Biometrika*, *95*(3), 759–771. https://doi.org/10.1093/biomet/asn034

Comtois, D. (2021). *summarytools: Tools to Quickly and Neatly Summarize Data*. https://CRAN.R-project.org/package=summarytools

Di Cook, T. N., McBain, M., & Fay, C. (2021). *naniar: Data Structures, Summaries, and Visualisations for Missing Data*. https://CRAN.R-project.org/package=naniar

Dowle, M., & Srinivasan, A. (2022). *data.table: Extension of `data.frame`*. https://CRAN.R-project.org/package=data.table

Epskamp, S. (2021). *graphicalVAR: Graphical VAR for Experience Sampling Data*. https://CRAN.R-project.org/package=graphicalVAR

Epskamp, S., Cramer, A. O. J., Waldorp, L. J., Schmittmann, V. D., & Borsboom, D. (2012). qgraph: Network Visualizations of Relationships in Psychometric Data. *Journal of Statistical Software*, *48*(4), 1–18.

Garnier, Simon, Ross, Noam, Rudis, Robert, Camargo, Antônio Pedro, Sciaini, Marco, Scherer, & Cédric. (2021). *viridis—Colorblind-Friendly Color Maps for R*. https://doi.org/10.5281/zenodo.4679424

Grolemund, G., Wickham, H., & Epskamp, S. (2021). Dates and Times Made Easy with lubridate: GraphicalVAR: Graphical VAR for Experience Sampling Data. *Journal of Statistical Software*, *40*(3), 1–25.

Klik, M. (2022). *fst: Lightning Fast Serialization of Data Frames*. https://CRAN.R-project.org/package=fst

Long, J. A. (2020). *jtools: Analysis and Presentation of Social Scientific Data*. https://cran.r-project.org/package=jtools

Mansueto, A. C., Wiers, R. W., van Weert, J. C. M., Schouten, B. C., & Epskamp, S. (2022). Investigating the feasibility of idiographic network models. *Psychological Methods*. https://doi.org/10.1037/met0000466

Moritz, S., & Bartz-Beielstein, T. (2017). imputeTS: Time Series Missing Value Imputation in R. *The R Journal*, *9*(1), 207–218. https://doi.org/10.32614/RJ-2017-009

Müller, K. (2020). *here: A Simpler Way to Find Your Files*. https://CRAN.R-project.org/package=here

Sali, A., & Attali, D. (2020). *shinycssloaders: Add Loading Animations to a “shiny” Output While It’s Recalculating*. https://CRAN.R-project.org/package=shinycssloaders

Wickham, H. (2016). *ggplot2: Elegant Graphics for Data Analysis*. Springer-Verlag New York. https://ggplot2.tidyverse.org

Wickham, H., François, R., Henry, L., & Müller, K. (2021). *dplyr: A Grammar of Data Manipulation*. https://CRAN.R-project.org/package=dplyr

Wilke, C. O. (2020). *cowplot: Streamlined Plot Theme and Plot Annotations for “ggplot2.”* https://CRAN.R-project.org/package=cowplot
